# Intrinsic Thermodynamics and Structure Correlation of Benzenesulfonamides with a Pyrimidine Moiety Binding to Carbonic Anhydrases I, II, VII, XII, and XIII

**DOI:** 10.1371/journal.pone.0114106

**Published:** 2014-12-10

**Authors:** Miglė Kišonaitė, Asta Zubrienė, Edita Čapkauskaitė, Alexey Smirnov, Joana Smirnovienė, Visvaldas Kairys, Vilma Michailovienė, Elena Manakova, Saulius Gražulis, Daumantas Matulis

**Affiliations:** 1 Department of Biothermodynamics and Drug Design, Institute of Biotechnology, Vilnius University, Graičiūno 8, Vilnius, LT-02241, Lithuania; 2 Department of Bioinformatics, Institute of Biotechnology, Vilnius University, Graičiūno 8, Vilnius, LT-02241, Lithuania; 3 Department of Protein – DNA Interactions, Institute of Biotechnology, Vilnius University, Graičiūno 8, Vilnius, LT-02241, Lithuania; Kermanshah University of Medical Sciences, Iran, Republic of Islamic

## Abstract

The early stage of drug discovery is often based on selecting the highest affinity lead compound. To this end the structural and energetic characterization of the binding reaction is important. The binding energetics can be resolved into enthalpic and entropic contributions to the binding Gibbs free energy. Most compound binding reactions are coupled to the absorption or release of protons by the protein or the compound. A distinction between the observed and intrinsic parameters of the binding energetics requires the dissection of the protonation/deprotonation processes. Since only the intrinsic parameters can be correlated with molecular structural perturbations associated with complex formation, it is these parameters that are required for rational drug design. Carbonic anhydrase (CA) isoforms are important therapeutic targets to treat a range of disorders including glaucoma, obesity, epilepsy, and cancer. For effective treatment isoform-specific inhibitors are needed. In this work we investigated the binding and protonation energetics of sixteen [(2-pyrimidinylthio)acetyl]benzenesulfonamide CA inhibitors using isothermal titration calorimetry and fluorescent thermal shift assay. The compounds were built by combining four sulfonamide headgroups with four tailgroups yielding 16 compounds. Their intrinsic binding thermodynamics showed the limitations of the functional group energetic additivity approach used in fragment-based drug design, especially at the level of enthalpies and entropies of binding. Combined with high resolution crystal structural data correlations were drawn between the chemical functional groups on selected inhibitors and intrinsic thermodynamic parameters of CA-inhibitor complex formation.

## Introduction

Significant effort has been devoted to develop drugs that bind to their targets with high affinity and sufficient selectivity [1], [2]. The binding affinity, *i.e*. the change in Gibbs free energy of binding (Δ*G*), is the sum of the changes in enthalpic and entropic contributions (Δ*H* and *T*Δ*S* respectively) that often compensate each other [3]. Optimization of the enthalpic and entropic contributions to binding is fundamental to improving the affinity of small molecule inhibitors in drug development. However, our capabilities to do this routinely are limited by our understanding of structure-thermodynamic correlations [4]–[9]. Thermodynamic measurement of the energetic contributions to protein-compound complex formation is not straightforward in the presence of additional contributions from solvent effects such as protonation/deprotonation of the interacting moieties, *i.e.* Δ*H* is the sum of the contributions from the Δ*H* of binding (intrinsic) and Δ*H* of protonation.

Thermodynamics has found increasing use in drug design and development when targeting the inhibition of carbonic anhydrases (CAs). CAs are zinc metal containing enzymes that catalyze the reversible hydration of CO_2_ and dehydration of bicarbonate. CAs perform important physiological functions in all kingdoms of life [10], [11]. There are 12 catalytically active CA isoforms in humans. CAs are involved in many physiological and pathological processes including pH and CO_2_ homeostasis, respiration and transport of bicarbonate and CO_2_ in various metabolizing tissues and lungs, electrolyte secretion, CO_2_ fixation and biosynthetic reactions, bone resorption, calcification and tumorigenicity [11]–[15]. Abnormal activities of CAs are often associated with different human diseases, such as glaucoma, epilepsy, Alzheimer’s and Parkinson’s diseases, obesity, and cancer [15]–[18]. Therefore CAs are important therapeutic targets and some inhibitors are clinically approved drugs [19]. The most studied class of CA inhibitors is aromatic sulfonamides [12], [20], [21]. Although about 30 CA inhibitors are currently used as drugs, the challenge of developing compounds that are selective for a specific isoform still remains [22], [23].

In this study the structure-thermodynamic profile of CA inhibitor binding was investigated. The underlying contributions of Δ*H* and *T*Δ*S* to the Δ*G* have been shown to be important parameters to integrate into rational drug design programs targeted at CAs [24], however, the directly measured values of these terms are non-intrinsic since they include the energetic contributions from protonation events that accompany the binding reaction between a CA and its compound [25], [26]. It is important to note that only the deprotonated form of the sulfonamide binds to the CA active site. Furthermore, the active site Zn-coordinated hydroxide must be protonated before it can be replaced by the amino group of the sulfonamide [27]. Therefore, the observed parameters depend on the conditions of the experiment, such as pH and buffer composition [28] and, therefore it is important to dissect the protonation-deprotonation contributions to the thermodynamic parameters of binding. Since the modification of functional groups is the basis of medicinal chemistry in rational drug development and is essential to optimization of a promising lead candidates, it is of fundamental importance to calculate the intrinsic parameters that can be used to estimate the effect of the addition or replacement of functional groups [29], [30].

Detailed investigation of the compound structure-activity relationships (SAR) is required in order to rationally design new compounds with desired properties [28], [31], [32]. Here we analyzed both the intrinsic thermodynamics of binding in terms of the compound chemical structure and also the structures of protein-ligand crystallographic complexes leading to a more-in-depth understanding of the binding reaction itself and the changes in binding profile as chemical modifications in drug-like molecules are made. Analysis of previously published structures of compounds bound to several CA isoforms [33], together with four newly solved crystal structures of CA II with compounds **1d**, **2c**, **4c**, and CA XIII with **4c**, revealed that all compounds bound to CAs in a similar mode but with significant differences that may be correlated to differences in the thermodynamics of binding. The series of 16 closely related compounds were analyzed and mapped in the direction of incrementally changing chemical functional groups to correlate with the increments in the intrinsic thermodynamic parameters. By determining the intrinsic thermodynamic binding parameters we are able to assess the important contributions to affinity and suggest routes to novel therapeutically useful compound development.

## Results and Discussion

### Observed thermodynamics of inhibitor binding to CA

**Figure 1:**
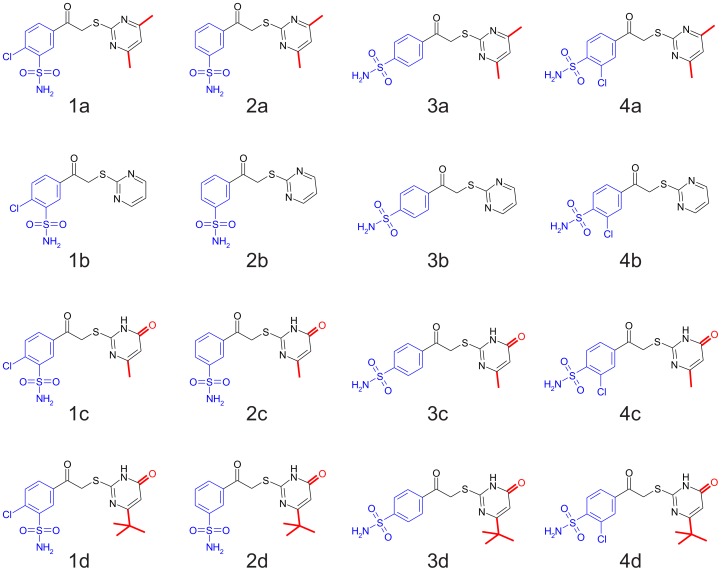
The structures of S-alkylated pyrimidine derivative compounds used in this study. Compounds are grouped according to 4 sulfonamide headgroups **(1–4)** and 4 tailgroups **(a–d)** totaling 16 compounds.

Sixteen compounds (**1a**, **2a**, …, **4d**, Fig. 1) made by combining four different aromatic sulfonamide headgroups (**1**, **2**, **3**, and **4**) with four modified pyrimidine tailgroups (**a**, **b**, **c**, and **d**) were chosen to study the correlations of compound chemical structure with the thermodynamics of binding. Furthermore, the energetic additivity of these head and tail-groups was investigated by comparing whether the addition of a particular tailgroup to different headgroups will contribute the same increment in thermodynamics of binding.

**Figure 2:**
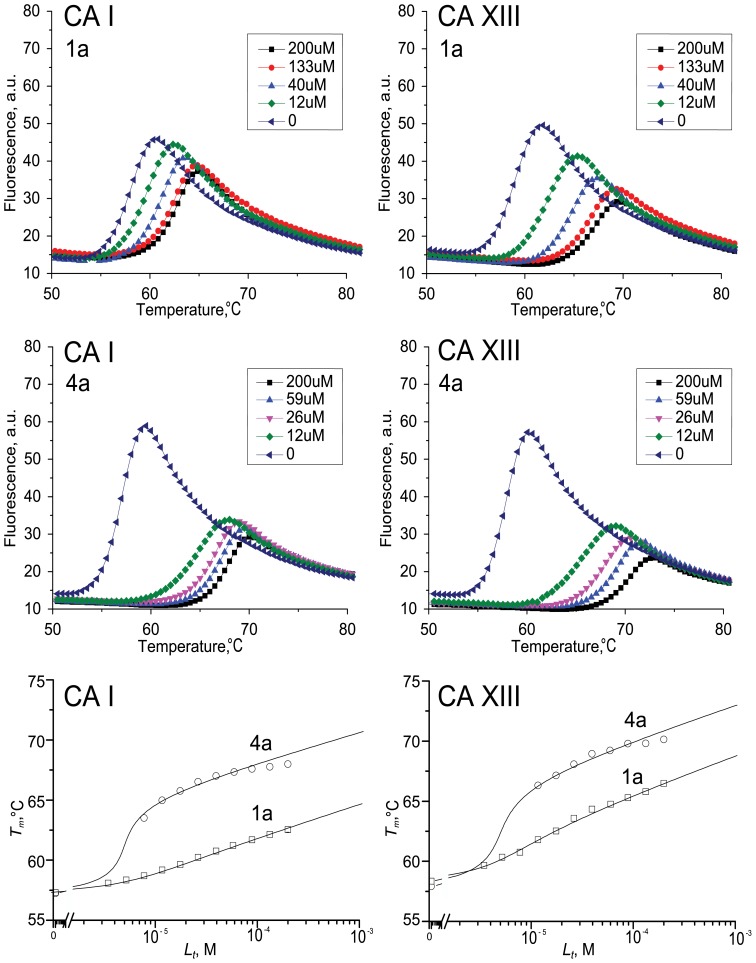
Fluorescent thermal shift assay data for selected compound 1a and 4a binding to CA I and CA XIII. Top graphs show raw protein denaturation curves and the bottom graphs show the compound dosing curves. Datapoints are the experimental midpoints of the denaturation curves while the lines are drawn according to the model [39].

**Figure 3:**
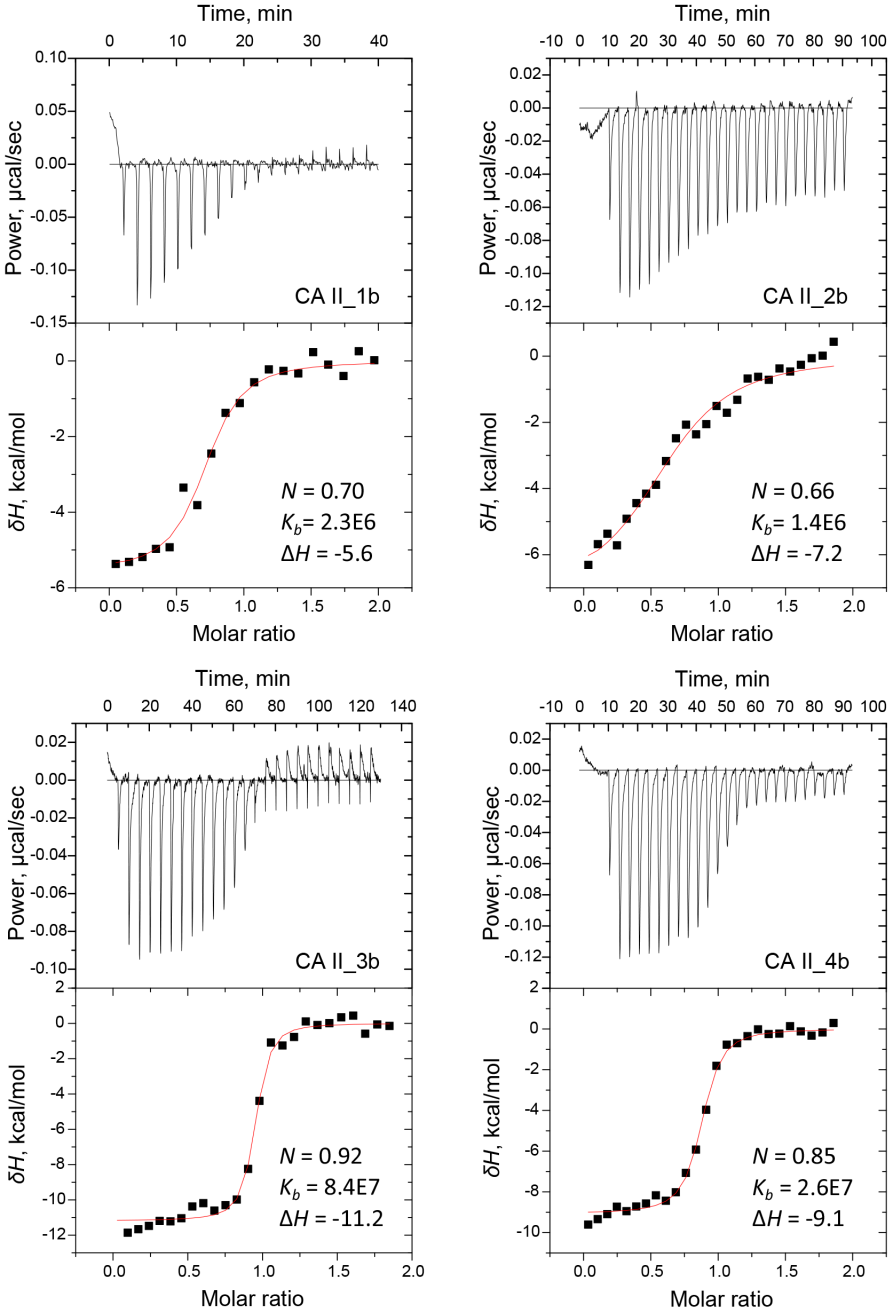
ITC data for compound (1–4)b binding to CA II. Upper graphs are power compensation curves while the lower graphs are the integrated ITC binding curves determined at 37°C.

**Figure 4:**
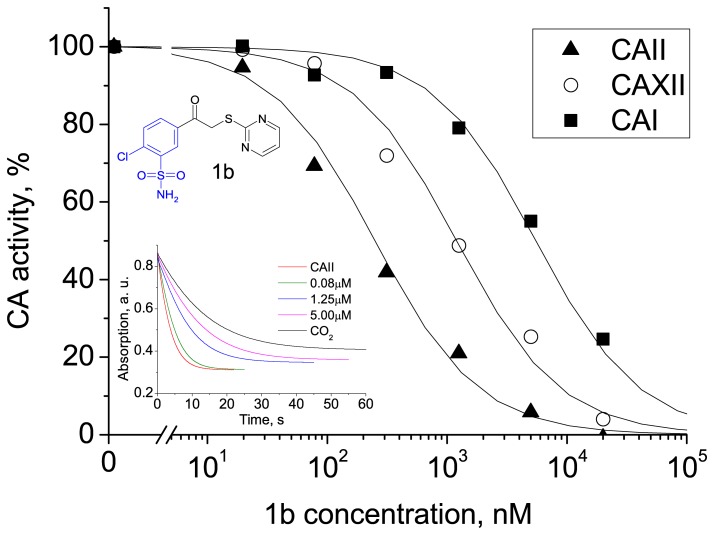
SFA data for inhibition of CA I, CA II and CA XII by compound 1b determined at 25°C. The inset shows the curves of pH drop monitored by indicator spectrophotometry for CA II.

The observed binding affinities (Δ*_b_G_obs_*) of the compounds were previously measured using fluorescent thermal shift assay (FTSA) as described by Čapkauskaite et al. [12], [33]. In this work, the binding of 16 inhibitors to five CA isoforms (CA I, CA II, CA VII, CA XII, and CA XIII) was analyzed using ITC which provided the observed enthalpy (Δ*_b_H_obs_*) and entropy (Δ*_b_S_obs_*) in addition to the Δ*_b_G_obs_*. The observed affinities determined by ITC essentially confirmed our previous FTSA results (Table 1). Furthermore, stopped-flow CO_2_ hydration assay (SFA) was used as control in order to confirm inhibition constants of compound **1b** binding to five isoforms of recombinant human carbonic anhydrases. The observed dissociation constants spanned the range from 0.25 nM to 10 µM. Figs. 2, 3 and 4 show typical experimental data of FTSA, ITC and SFA measurements. The observed enthalpies and entropies of binding, determined by ITC, are listed in Table 1.

**Table 1:** Experimentally measured observed dissociation constants (determined by FTSA and ITC), averaged Gibbs free energies, and the enthalpies and entropies (determined by ITC) of the compound binding to five isoforms of human recombinant CAs.

CA I		1a	1b	1c	1d	2a	2b	2c	2d	3a	3b	3c	3d	4a	4b	4c	4d
	*K_d_ITC_*	850	2300	2000	240	100	1500	1900	230	85	110	180	23	19	49	90	7.0
	*K_d_FTSA_*	1000	3100	830	100	370	770	830	100	22	67	67	1.4	5.0	10	4.0	0.25
	Δ*G*	−35.8	−33.1	−35.0	−40.4	−36.9	−35.4	−35.0	−40.5	−43.7	−41.9	−41.3	−49.0	−47.6	−45.5	−45.9	−52.7
	Δ*H*	−18.1	−16.6	−18.3	−26.0	−33.2	−31.7	−24.3	−26.2	−31.3	−27.9	−23.3	−37.4	−29.2	−28.6	−20.8	−28.1
	*T*Δ*S*	17.7	16.5	16.7	14.4	3.7	3.7	10.7	14.3	12.4	14.0	18.0	11.6	18.4	16.9	25.1	24.6
**CA II**	***K_d_ITC_***	150	430	130	100	570	730	1100	740	64	12	49	27	14	38	20	90
	***K_d_FTSA_***	220	330	200	50	1000	1100	1300	500	33	17	67	45	17	11	21	40
	Δ***G***	−40.0	−38.1	−40.4	−42.4	−36.4	−35.9	−35.2	−36.9	−43.6	−46.6	−43.0	−44.2	−46.4	−45.6	−45.6	−42.9
	Δ***H***	−14.0	−23.3	−19.4	−22.9	−22.6	−30.1	−14.2	−12.3	−33.2	−46.8	−22,6	−19.5	−31.4	−37.9	−19.4	−12.4
	***T***Δ***S***	26.0	14.8	21.0	19.5	13.8	5.8	21.0	24.6	10.4	−0.2	20.4	24.7	15.0	7.7	26.2	30.5
**CA VII**	***K_d_ITC_***	460	660	320	92	10000	1600	12000	3500	260	82	630	340	61	89	68	230
	***K_d_FTSA_***	330	710	130	40	6700	4000	4000	500	130	67	170	33	13	5,0	36	11
	Δ***G***	−38.0	−36.6	−39.7	−42.9	−30.2	−33.2	−30.7	−30.7	−40.1	−42.3	−38.5	−41.4	−44.9	−45.6	−43.4	−43.3
	Δ***H***	−23.6	−44.7	−17.0	−23.7	−10.4	−10.2	−10.6	−4.6	−54.3	−50.7	−54.3	−27.4	−19.4	−31.6	−14.6	−15.7
	***T***Δ***S***	14.4	−8.1	22.7	19.2	19.8	23.0	20.1	26.1	−14.2	−8.4	−15.8	14.0	25.5	14.0	28.8	27.6
**CA XII**	***K_d_ITC_***	1400	370	550	84	7900	620	2800	940	230	370	330	29	460	450	500	NA
	***K_d_FTSA_***	1400	1300	500	43	3300	3300	3700	1300	910	560	560	140	910	830	330	330
	Δ***G***	−34.8	−36.6	−37.3	−42.9	−31.4	−34.7	−32.6	−35.4	−37.6	−37.7	−37.8	−42.7	−36.7	−36.9	−38.5	−38.5
	Δ***H***	−18.2	−19.4	−10.8	−9.0	−28.4	−15.5	−12.6	−13.4	−23.4	−30.7	−11.8	−10.8	−29.1	−24.7	−24.7	NA
	***T***Δ***S***	16.6	17.2	26.5	33.9	3.0	19.2	20.0	22.0	14.2	7.0	26.0	31.9	7.6	12.2	13.8	NA
**CA XIII**	***K_d_ITC_***	300	270	88	62	23000	700	3500	2100	130	77	1500	50	25	44	144	5.6
	***K_d_FTSA_***	330	330	200	25	1000	1300	1500	170	83	130	360	5.0	10	17	33	5.0
	Δ***G***	−38.6	−38.7	−40.8	−44.0	−31.6	−35.7	−33.4	−37.0	−41.4	−41.6	−36.4	−46.3	−46.3	−44.9	−42.5	−49.2
	Δ***H***	−11.9	−15.4	−16.5	−20.9	−27.9	−16.0	−14.8	−27.7	−35.9	−33.9	−29.1	−24.9	−33.9	−37.1	−23.8	−26.9
	***T***Δ***S***	26.7	23.3	24.3	23.1	3.7	19.7	18.6	9.3	5.5	7.7	7.3	21.4	12.4	7.8	18.7	22.3

Experimental results are for 37°C, pH 7.0. Intrinsic binding parameters were calculated from these data using Eq. (1–4) and are listed in table 3.

NA – not determined by ITC due to poor solubility of the compound and relatively high concentration due to weak binding as compared to other CA isoforms.

### Thermodynamics of protonation of the inhibitor and zinc-bound hydroxide anion

The sulfonamide group of the inhibitor must be deprotonated in order to bind to CA [27]. For calculations of intrinsic parameters, it is important to know the enthalpy of deprotonation of sulfonamide (Δ*H_prot_*) and its p*K_a_* value. The p*K_a_* was calculated and measured experimentally. For compounds containing chlorine (**1**(**a–d**) and **4**(**a–d**)**)**, the calculated p*K_a_* values were equal to 8.7 (Table 2). For inhibitors without chlorine (**2**(**a–d**) and **3**(**a–d**)), the calculated p*K_a_* values were equal to 9.7. The difference between the calculated values of compounds **a**, **b**, **c**, and **d** did not exceed ±0.2.

**Table 2:** p*K_a_* values and Δ*H_prot_* of compounds (**1**–**4**)**b** and the p*K_a_* values and Δ*H_prot_* of H_2_O bound to Zn in the active site of human recombinant CAs at 37°C.

Compound	p*K_a_*(calculated)	p*K_a_*(measured)	Δ*H_prot_*,kJ/mol
**1b**	8.7	8.9	−24.3
**2b**	9.7	9.4	−29.7
**3b**	9.7	9.4	−29.7
**4b**	8.7	8.9	−24.3
**CA**			
**CAI-Zn-H_2_O**	–	8.1^a^	−38.5^a^
**CAII-Zn-H_2_O**	–	6.9^a^	−23.5^a^
**CAVII-Zn-H_2_O**	–	6.8^b^	−30.5^b^
**CAXII-Zn-H_2_O**	–	6.8^c^	−25.5^c^
**CAXIII-Zn-H_2_O**	–	8.0^d^	−41.5

a from [37], ^b^from [36], ^c^from [35], ^d^from [31].

**Figure 5:**
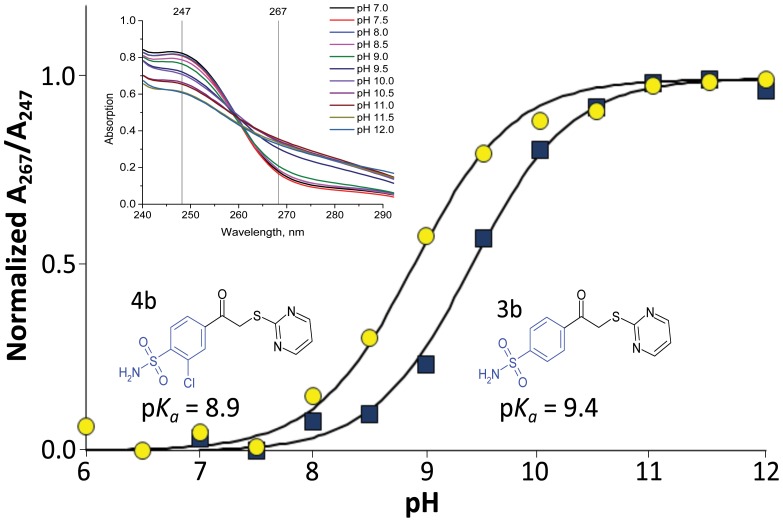
Spectrophotometric determination of the sulfonamide group deprotonation p*K_a_* for 3b and 4b. The inset shows the absorption spectra of compound **3b** in solutions at various pH.

The p*K_a_* values were also determined spectrophotometrically for compounds (**1**–**4**)**b** according to [34] as described in materials and methods. The absorbance spectra of the four compounds were taken at various pH (Fig. 5). The ratio of absorbance at two wavelengths where the pH effect is large (10 nm above and below isosbestic point) was normalized and plotted as a function of pH yielding the p*K_a_* of the sulfonamide headgroup. For compounds **1**(**a–d**) and **4**(**a–d**) containing Cl in the benzene ring, the experimental p*K_a_* was 8.9±0.2 and for compounds **2**(**a–d**) and **3**(**a–d**) the experimental p*K_a_* was 9.4±0.2. These values are similar to the calculated values. Since compound **a**, **c**, and **d** solubility at high pH prevented accurate p*K_a_* determination, and as confirmed by calculation, we assumed that compounds **a**, **c**, and **d** would have the same p*K_a_*s and therefore these values were later used in calculations of intrinsic parameters (Table 3).

**Table 3:** Intrinsic dissociation constants, Gibbs free energies, enthalpies, and entropies of the compound binding to five isoforms of human recombinant CAs.

		1a	1b	1c	1d	2a	2b	2c	2d	3a	3b	3c	3d	4a	4b	4c	4d
**CA I**	***K_d_***	11	31	15	1.8	2.2	4.0	4.7	0.55	0.16	0.32	0.41	0.021	0.11	0.26	0.015	0.22
	**Δ** ***G***	−47.3	−44.6	−46.5	−51.9	−51.4	−49.9	−49.5	−55.0	−58.2	−56.4	−55.8	−63.4	−59.1	−57.0	−64.3	−57.4
	**Δ** ***H***	−36.6	−35.1	−36.8	−44.5	−57.3	−55.8	−48.4	−50.3	−55.4	−52.0	−47.4	−61.5	−47.7	−47.2	−46.7	−39.4
	***T*** **Δ** ***S***	10.7	9.5	9.7	7.4	−5.9	−5.9	1.1	4.7	2.8	4.4	8.4	1.9	11.4	9.8	17.6	18.0
**CA II**	***K_d_***	1.0	2.1	0.88	0.40	1.3	1.6	2.1	1.1	0.08	0.025	0.10	0.062	0.085	0.11	0.11	0.33
	**Δ** ***G***	−53.5	−51.6	−53.8	−55.8	−52.7	−52.3	−51.6	−53.3	−59.9	−63.0	−59.4	−60.6	−59.8	−59.0	−59.0	−56.3
	**Δ** ***H***	−23.7	−33.0	−29.1	−32.6	−37.8	−45.3	−29.5	−27.5	−48.4	−62.0	−37.8	−34.7	−41.1	−47.6	−29.0	−22.0
	***T*** **Δ** ***S***	29.8	18.6	24.7	23.2	14.9	7.0	22.1	25.8	11.5	1.0	21.6	25.9	18.7	11.4	30.0	34.3
**CA VII**	***K_d_***	1.9	3.3	1.0	0.29	12	3.9	10	2.0	0.27	0.11	0.50	0.16	0.13	0.10	0.24	0.24
	**Δ** ***G***	−51.8	−50.4	−53.4	−56.6	−46.9	−49.9	−47.4	−45.5	−56.8	−59.0	−55.2	−58.1	−58.7	−59.3	−57.2	−57.1
	**Δ** ***H***	−27.8	−48.9	−21.2	−27.9	−20.2	−19.9	−20.4	−14.4	−67.3	−60.4	−64.1	−37.2	−23.6	−35.8	−18.8	−19.9
	***T*** **Δ** ***S***	24.0	1.5	32.3	28.7	26.7	30.0	27.0	31.1	−10.5	−1.4	−8.9	20.9	35.1	23.5	38.4	37.2
**CA XII**	***K_d_***	6.7	3.3	32	0.29	7.8	2.2	4.9	1.7	0.71	0.70	0.65	0.10	3.1	3.0	2.0	1.6
	**Δ** ***G***	−48.5	−50.4	−51.0	−56.6	−48.1	−51.4	−49.3	−52.1	−54.3	−54.4	−54.5	−59.4	−50.5	−50.6	−51.7	−52.2
	**Δ** ***H***	−25.5	−26.7	−18.1	−16.3	−41.3	−28.3	−25.4	−26.2	−36.3	−43.5	−24.6	−23.6	−36.4	−32.0	−19.1	NA
	***T*** **Δ** ***S***	23.0	23.7	32.9	40.3	6.8	23.1	23.9	25.9	18.0	10.9	29.9	35.8	14.1	18.6	32.6	NA
**CA XIII**	***K_d_***	3.6	3.4	1.5	0.44	17	3.5	8.4	2.2	0.38	0.35	2.6	0.057	0.18	0.31	0.78	0.060
	**Δ** ***G***	−50.2	−50.3	−52.4	−55.5	−46.1	−50.2	−47.9	−51.5	−56.0	−56.1	−51.0	−60.8	−57.9	−56.5	−54.1	−60.7
	**Δ** ***H***	−29.5	−33.0	−34.1	−38.6	−51.1	−39.3	−38.0	−50.9	−59.1	−57.1	−52.3	−48.1	−51.6	−54.8	−41.4	−44.5
	***T*** **Δ** ***S***	20.7	17.3	18.3	16.9	−5.0	10.9	9.9	0.6	−3.1	−1.0	−1.3	12.7	6.3	1.7	12.7	16.2

NA – not determined by ITC due to poor solubility of the compound and relatively high concentration due to weak binding as compared to other CA isoforms.

**Figure 6:**
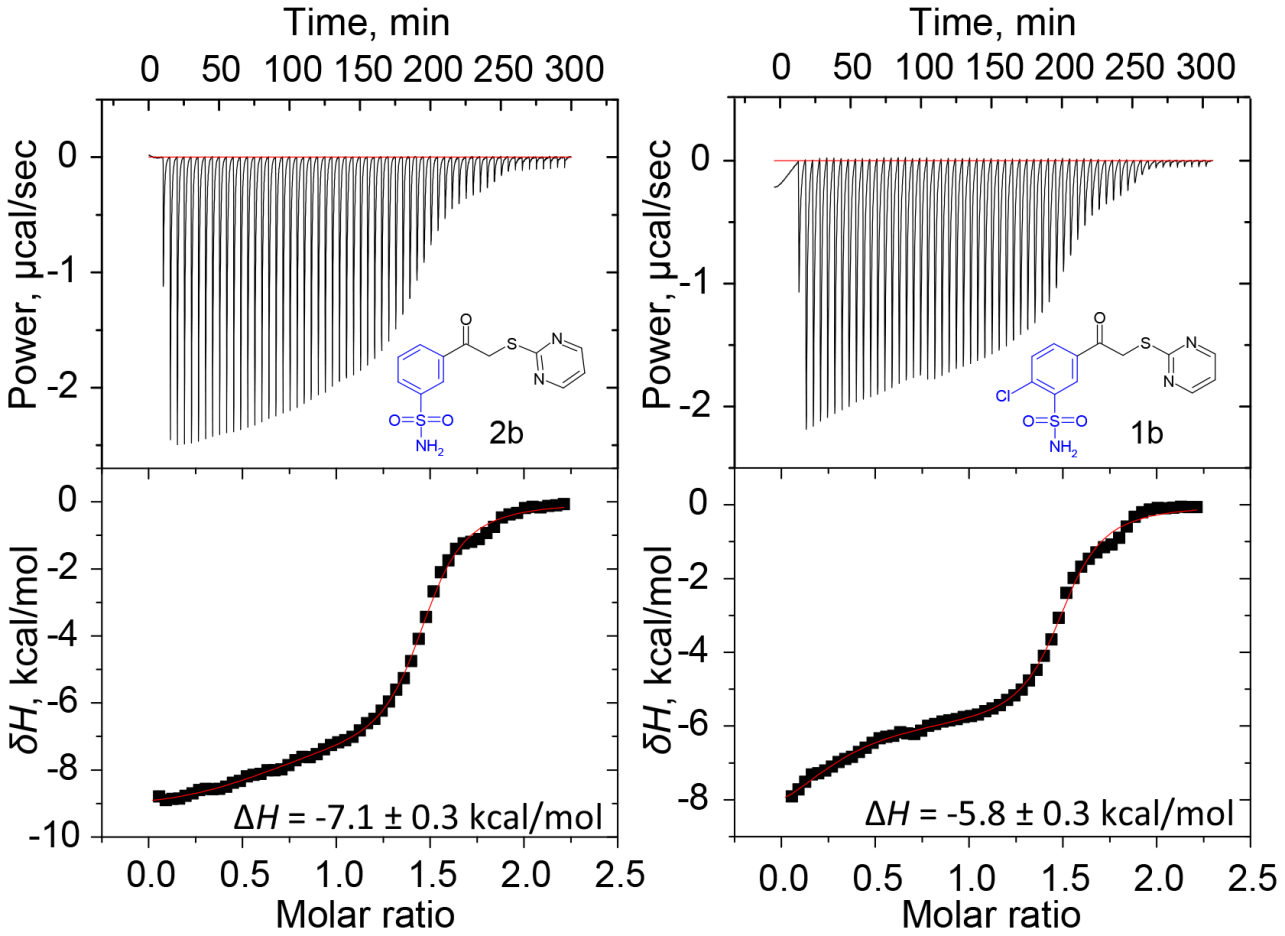
Determination of the enthalpy of protonation for 1b and 2b by ITC. Compounds were deprotonated by adding 1.5 equivalent NaOH and titrating with HNO_3_.

The Δ*H_prot_* of sulfonamide group for compounds (**1**–**4**)**b** was determined using ITC (Table 2). First, the inhibitor was deprotonated by applying 1.5 equivalent NaOH to reach the pH at least one unit above the p*K_a_*. Then the deprotonated inhibitor solution was titrated with acid by ITC. Titration curves of **1b** and **2b** are shown in Fig. 6. The first transition represents the titration of excess NaOH, the second transition represents the titration of inhibitor. The inhibitors with chlorine (**1b** and **4b**) had protonation enthalpy of −5.8±0.3 kcal/mol (−24.3 kJ/mol) while the inhibitors without chlorine (**2b** and **3b)** exhibited the enthalpies of protonation −7.1±0.3 kcal/mol (−29.7 kJ/mol) (Table 2). The effect of modifications in pyrimidine ring for Δ*H_prot_* values was below the standard error of the measurement.

The Zn-bound hydroxy ion in the active site of CA must be protonated for the binding reaction to occur. Thermodynamic parameters of protonation of the hydroxide in the five CA isoforms were previously determined using ITC for 25°C [31], [35]–[37]. In Table 2, the p*K_a_* and Δ*H_prot_* values for all investigated CA isoforms were calculated for 37°C. CA I has similar p*K_a_* and Δ*H_prot_* values to CA XIII, CA II has similar p*K_a_* value to CA VII and CA XII, and similar Δ*H_prot_* value to CA XII. Some of these similarities can be explained by comparing amino acids in the active site of CA isoforms.

### Intrinsic thermodynamics of inhibitor binding

**Figure 7:**
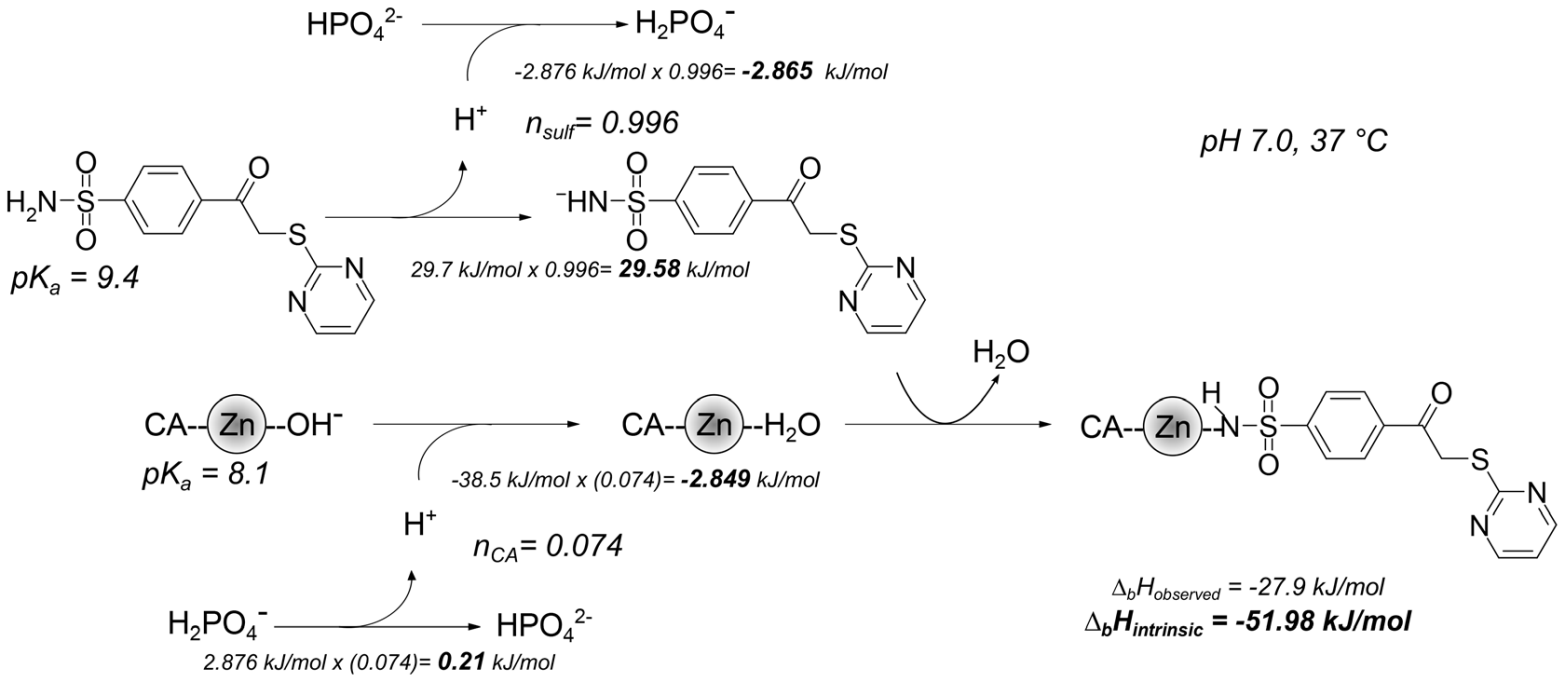
The processes linked to the binding of 3b to CA I. The two left-central reactions show the binding-linked deprotonation of the inhibitor sulfonamide and the protonation of the zinc-bound hydroxide, respectively. Top and bottom lines show linked phosphate buffer (de)protonation reactions. The numbers give estimates of the enthalpies for each process multiplied by the number of linked protons (n) yielding the observed enthalpic contribution of each reaction at pH 7.0, 37°C. The intrinsic enthalpy of binding, shown by the rightmost arrow, is equal to −51.98 kJ/mol. The observed enthalpy, estimated for phosphate buffer at pH 7.0, is equal to −27.90 kJ/mol. Zinc atom is shown as grey shaded sphere and the carbonic anhydrase protein is shown as CA.

Fig. 7 shows a scheme of the processes linked to the binding of **3b** to CA I. The p*K_a_* of CA I is 8.1 and Δ*_b_proton_CA_H* value is −38.5 kJ/mol. The p*K_a_* value of **3b** is 9.4 and Δ*_b_proton_sulf_ H* value is −29.7 kJ/mol. The enthalpy of phosphate buffer protonation Δ*_b_proton_buf_ H* = −2.9 kJ/mol. *f_CA_* = 0.926, *f_sulf_* = 0.004. *n_sulf_* = 1 − *f_sulf_* = 0.996, *n_CA_* = 1 *− f_CA_* = 0.074 and *n_buf_ = n_sulf_+n_CA_*. From equation 4 (materials and methods), the intrinsic enthalpy is Δ*_b_H* = −52.0 kJ/mol. All compounds binding intrinsic parameters are listed in Table 3.

### Compound structure correlations with binding thermodynamics

**Figure 8:**
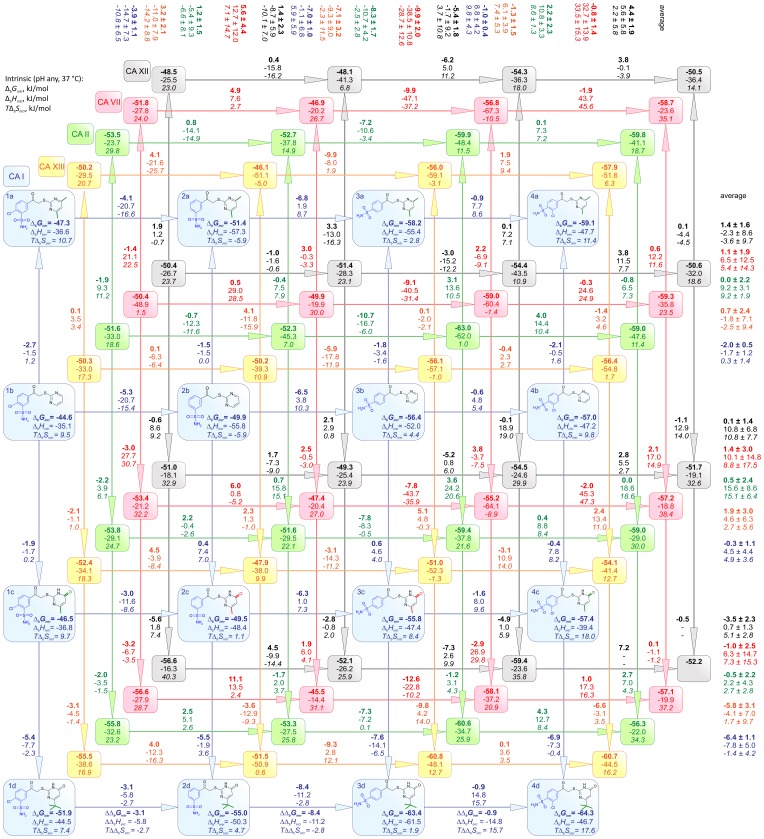
Inhibitor structure correlations with the thermodynamics of binding. Intrinsic parameters of compound binding to five investigated CA isoforms are given within the shapes. Different colors represent different CA isoforms. Numbers next to arrows show the Gibbs free energy (top number, bold), enthalpy (middle number), and entropy (*T*Δ*_b_S*, bottom number) of binding differences between two neighboring compounds (in kJ/mol at 37°C). Numbers to the top and right of the map are averages between same heads and tails of the compounds. The standard deviations indicate the presence and absence of the energetic additivity of compound functional groups.

The intrinsic thermodynamic parameters 16 compounds binding to five CA isoforms are shown in Fig. 8. The map shows inhibitor structure correlations with the thermodynamics of binding. The most similar compounds are arranged next to each other and the arrows show which compounds are being compared. The intrinsic Δ*_b_G* (top number, bold), Δ*_b_H* (middle number), and *T*Δ*_b_S* (bottom number, itallic) values for binding are listed within the shapes and the values next to the arrows show the incremental differences in thermodynamic values between the two compounds. The incremental differences show how much energy is gained or lost when adding or removing a chemical group of the compound.

Different colors on the map represent different CA isoforms, blue – CA I, yellow – CA XIII, green – CA II, red – CA VII, and black – CA XII. The order of the isozymes is shown not according to the numbering of CAs, but according to the similarities of the active site of the protein. To determine the similarity between the active sites, the following amino acids were considered to be important (numbers of CA II in UniProt database): 62, 64, 65, 67, 91, 121, 130, 134, 140, 197, 199, 201, and 203. These amino acids were compared among the five CAs and it was determined that the similarity between CA I and CA II is 30.8% while the similarity between CA I and CA XIII is 46.2%. CA XIII is quite similar to CA II (61.5%). The arrangement of CAs is according to the similarity of the active site, but it is quite arbitrary because it depends on which amino acids are considered important for the recognition of the compound.

The intrinsic Δ*_b_G* of CA I binding is more negative in the direction **1** → **2** → **3** → **4**. Therefore, the *para* position for the sulfonamide is better than *meta* in terms of affinity. Comparing **3** → **4**, the affinity changes slightly (1 kJ/mol), but Δ*_b_H* and Δ*_b_S* contributions change significantly more. For the inhibitors of group **3**, the binding to CA is driven by enthalpy. When chlorine is incorporated (group **4**), Δ*_b_H* favorable contribution decreases by 8.8 kJ/mol, while *T*Δ*_b_S* favorable contribution increases by 9.8 kJ/mol. However, when compound structure changes in direction **2** → **1** (incorporation of chlorine in the *para* position), the binding affinity increases by 3.9 kJ/mol, but the Δ*_b_H* contribution becomes less favorable by 14.7 kJ/mol and *T*Δ*_b_S* contribution increases by 10.8 kJ/mol. The incorporation of chlorine both in *meta* and *para* substituted compounds makes entropy more favorable while the enthalpy less favorable. It can be explained by the hydrophobicity of the chlorine. Comparing **2** → **3** (change in the position of sulfonamide headgroup from *meta* to *para*), Δ*_b_G* becomes more favorable by 7 kJ/mol. In groups **a**, **b**, and **c**, the change occurs due to more favorable entropy (6.5 kJ/mol), while in group **d**, the enthalpic contribution is greater than entropic. For all inhibitors in groups **2** and **3**, the binding to CA I is enthalpy driven (entropy is negative in some cases). The Δ*_b_G* of compound binding to CA I is more negative in the direction **a** → **b** → **c** → **d**, though Δ*_b_G* of **b** → **c** modification has a value of approximately zero, thus the incorporation of oxo and methyl groups did not affect the binding affinity. The strongest binders for CA I are **3d** and **4d**.

**Figure 9:**
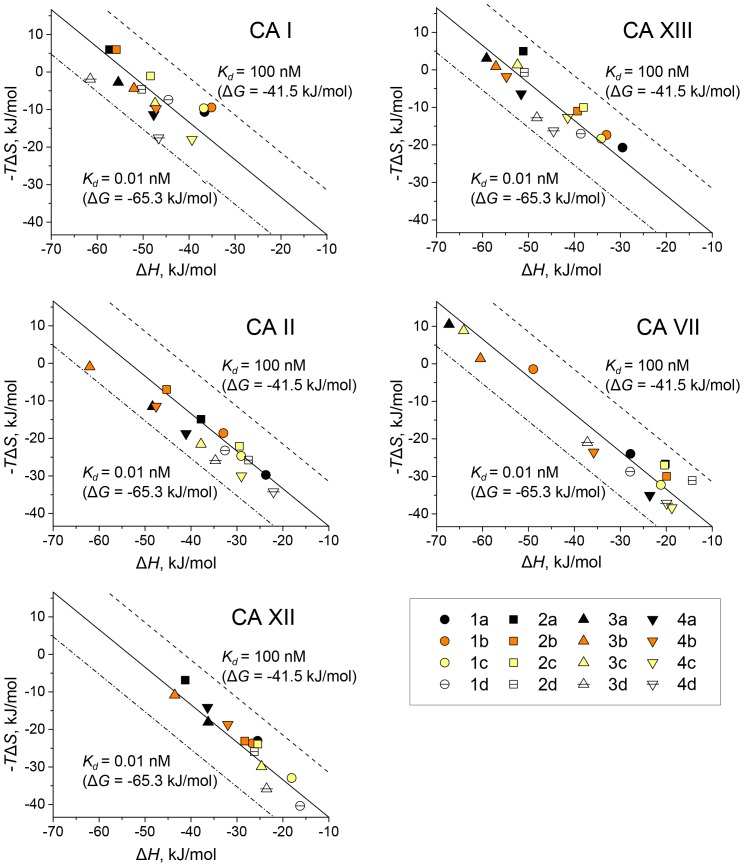
The intrinsic enthalpy – entropy compensation graphs for the 16 studied compound binding to five CA isoforms. Diagonal central line represents the dissociation constant of 1.0 nM (Δ*_b_G* = −53.4 kJ/mol at 37°C) while adjacent dashed lines show extremely tight binding of 10 pM or lower affinity at 100 nM. The compounds exhibit the range of affinities between 0.01 and 10 nM (Δ*_b_G* between −65.3 and −47.5 kJ/mol) while the enthalpies varied to a greater extent between −14 and −68 kJ/mol. The entropies (*T*Δ*_b_S*) also span the wide range between 11 and −41 kJ/mol.

Fig. 9 shows the enthalpy – entropy compensation graphs for the 16 compounds binding to the five CAs. Affinities of the compounds (Δ*_b_G*) span relatively narrow range as compared to the range of enthalpies and entropies. Some compounds exhibit strongly enthalpy-driven binding, while others of very similar chemical structure have significant entropic contribution to binding.

The compound binding affinities to CA XIII (Figs. 8 and 9) increase in the direction **2** → **1** and **2** → **3** → **4**. The Δ*_b_G* change in the direction **3** → **4** (incorporation of chlorine) is relatively small, but the enthalpy becomes less favorable while the entropy – more favorable. The same energetic changes happen when chlorine is incorporated in *meta* substituted inhibitors. In direction **2** → **3** (change in the position of sulfonamide headgroup from *meta* to *para*), the Δ*_b_G* decreases by 7.1 kJ/mol. The **2** → **3** change in groups **a**, **b**, and **c** has more favorable enthalpy (13.4 kJ/mol more negative) and in group **d**, contribution of enthalpy decreases. The binding entropy of **3a**, **3b**, and **3c** to CA XIII is unfavorable and it is enthalpy-driven. Incorporation of oxo and methyl or dimethyl groups does not affect the binding affinity, but modification of tert-butyl in pyrimidine ring changes Δ*_b_G* to more negative by 5.8 kJ/mol. The strongest binders for CA XIII are inhibitors **3d** and **4d** and similarly to CA I, the reaction is enthalpy-driven.

For CA II, the binding affinity increases in the direction **2** → **3** (8.3 kJ/mol), but for **1** → **2** and **3** → **4** it slightly decreases. Though Δ*_b_G* changes are small, for the changes **2** → **1** and **3** → **4**, the entropy becomes more favorable while the enthalpy – less favorable. However, for **2** → **3**, the enthalpy becomes more favorable with a minor change in entropy. For modifications **3b** → **3c** and **4b**→ **4c**, the entropy increases on average by 19.6 kJ/mol. This can be explained by a higher residual mobility of group **c** compounds in active site of CA II.

The modifications **3b** → **3a** and **4b** → **4a** also increase the entropy of binding, but not so strongly (8.9 kJ/mol). Similar to CA I and CA XIII, the incorporation of oxo and methyl or dimethyl groups does not affect the binding affinity to CA II. However, differently from CA I and CA XIII, the incorporation of tert-butyl in pyrimidine ring does not change the binding affinity to CA II. The strongest binders for CA II are inhibitors **3b** and **3d** and they are also enthalpy-driven.

For CA VII, the binding affinity increases in the direction of **2** → **3** (9.9 kJ/mol). In **1** → **2**, the binding affinity decreases while in **3** → **4**, there are no visible changes. For **3** → **4**, the enthalpy becomes less favorable (by 32.7 kJ/mol) while the entropy – more favorable (by 33.5 kJ/mol). Here we can see nicely visible entropy – enthalpy compensation phenomenon. In contrast from CA I, CA II, and CA XIII, here for **2** → **1**, the enthalpy becomes more favorable but entropy – less favorable. For **2** → **3**, the contribution of enthalpy is very high (change of 38.5 kJ/mol), while **b** → **a** and **b** → **c** → **d** has no effect on the binding affinity. CA VII exhibits high affinity with all inhibitors **3** and **4**, where the strongest binders are **3b** and **4b**. Binding of **3b** and **4b** is enthalpy-driven, but there are compounds that exhibit greater portion of entropy driven binding with CA VII.

For CA XII, the binding affinity increases only in direction **2** → **3** (by 5.4 kJ/mol). In **1** → **2** and **3** → **4**, it slightly decreases. Although Δ*_b_G* changes are small, for **1** → 2 and **3** → **4**, the entropy becomes more while enthalpy – less favorable. Incorporation of oxo and methyl or dimethyl groups does not affect the binding affinity, but incorporation of tert-butyl in the pyrimidine ring changes Δ*_b_G* to more negative by 3.5 kJ/mol. The strongest binders for CA XII are **1d** and **3d** and they exhibit greater portion of entropy driven binding.

S1 Figure shows the compound chemical structure correlation map with the intrinsic and observed binding affinities (*K_d_*). For structure – thermodynamics considerations, it is important to have intrinsic binding values. However, in order to know the affinities that are observed in practice and have the contributions of real fractions at pH 7.0, one may use the observed affinities at pH 7.0, that were determined both by FTSA and ITC, and the experimental values are listed in Table 1.

### Protein-compound crystallographic structure correlations with binding thermodynamics

**Figure 10:**
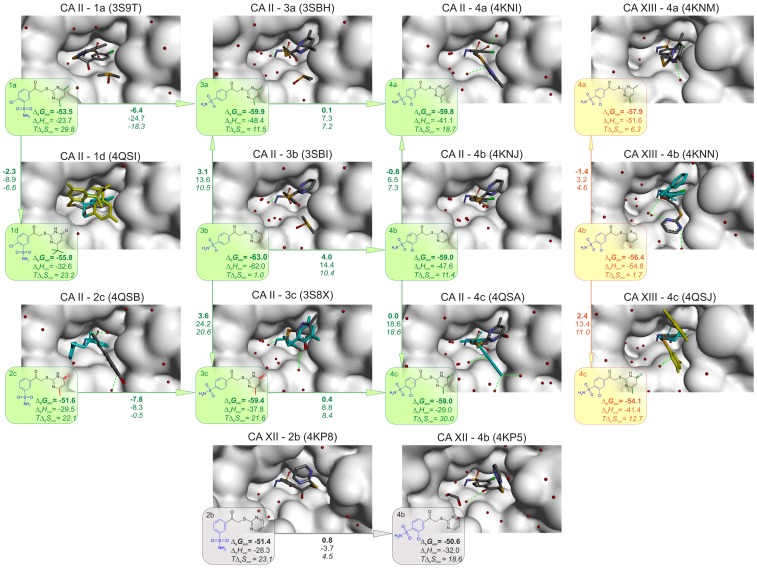
Compound chemical structure and the thermodynamics of binding correlations with the crystal structures of some compound binding mode in the active site of CAs (1a, 1d, 3a, 3b, 3c, 4a, 4b, and 4c with CAII; 4a, 4b, and 4c with CA XIII; 2b and 4b with CA XII). The thermodynamic parameters of binding and the colors of the shapes are same as in Figure 8 and indicate the CA isoform. Colors in the crystal structures are: yellow shows the pyrimidine ring that is not fixed in the crystal structure and has multiple conformations with low occupancies; blue shows the alternative conformation of the pyrimidine ring when both conformations are visible in the electron density maps.

Fig. 10 shows the 14 CA-compound complex crystal structures that were solved with this series of compounds. Nine structures are available for CA II, 3– for CA XIII, and 2– for CA XII. The PDB IDs and the thermodynamic values of binding are shown next to the structurally aligned protein surface views showing the main mode of compound binding and the position of water and cosolvent molecules. All compounds bound to CA by making a coordination bond between the sulfonamide nitrogen and protein Zn atom. The benzene ring containing the sulfonamide headgroup is clearly visible in the electron density of all structures. However, the second ring is partially or mostly flexible and poorly visible in some structures. Two possible orientations of the second ring are visible in some structures.

Comparison of the crystal structures of **1a** and **1d** bound to CA II (Fig. 10, 3S9T and 4QSI, respectively) shows that the Cl-benzene ring in both cases is positioned similarly, with the Cl in the hydrophobic pocket. The first ring of **1d** - CA II is well defined in the crystal structure, but the electron density of the acetylthio linker could not be interpreted unambiguously and therefore two alternative positions of the linker are modeled placing sulfur atoms into the electron density blobs. There is no electron density for the pyrimidine rings, their positions are deduced from the positions of linkers (see also S2 Figure, A). Therefore, the pyrimidine ring is not fixed in the crystal structure and has multiple conformations with low occupancies. However, entire structure of the inhibitor **1a** (3S9T, Fig. 10) could be fit to the electron density.

Inhibitors **3a**, **3b**, and **3c** in the active center of CA II (3SBH, 3SBI, and 3S8X) are bound in a similar way and differ only in the conformations of linkers between the pyrimidine and benzene rings. The same situation was observed while comparing **4a**, **4b**, and **4c** bound to CA II (4KNI, 4KNJ, and 4QSA). Despite different location of the Cl-benzene (**4**(**a**–**c**)), which is found in two alternative conformations in these structures, and benzene (**3**(**a**–**c**)) rings, the pyrimidine moiety is located in the same position between Phe131 and Pro202, except for **4c** and **4a**, where it is found on the other side relative to the Phe131. Compound **4c** in CA II is found in alternative conformations (S2 Figure, C). The pyrimidine rings are located on both sides of Phe131 and Cl-benzene is also in two orientations.

Compounds of group **a** are well visualized in the crystal structures of **1a**, **3a**, and **4a** in the active centers of CA II (3S9T, 3SBH, and 4KNI) and **4a** bound in CA XIII (4KNM). The position of the Cl-benzene ring in crystal structures of groups **1** and **4** is more variable (Cl atom positions in these structures coincide except for CA II –**4c**, the planes of the rings vary by up to 2.1 Å). In contrast, the benzene rings of *para*-substituted group **3** (3SBH, 3SBI, and 3S8X) nearly coincide with each other. Pyrimidine rings also coincide within this group. *Meta*-substituted group **2** in the active center of CA II is represented by **2c** (4QSB, S2 Figure, B). The benzene ring in the crystal structure is found in two alternative conformations, shifted with respect to the position of the benzene ring of group **3**. A possible explanation could be a clash of the *meta*-substituent of group **2** either with Phe131, or with Pro202. The pyrimidine rings are found in two positions (S2 Figure, B) and neither is similar to group **3**, poorly resolved in the crystal structure. It is interesting to compare the binding of **2c** in CA II with the crystal structure of **2b** in CA XII (4KP8). In the active center of CA XII, the benzene as well as pyrimidine rings are found in similar positions to **3** compounds in CA II, where the differences between *meta* and *para* position of pyrimidine are compensated by linker conformation. The absence of Phe131 in CA XII makes such orientation of the linker possible.

Crystal structures of CA XII with **4b** and **2b** exhibit different orientations of the headgroup benzene rings. The pyrimidine rings are positioned similarly, but the plane orientation is different. There are no steric hindrance issues for the *meta*-substituted **2b** in CA XII, as it would be expected for CA II due to the absence of Phe131. For **2b** → **4b**, the formation of a hydrogen bond between inhibitor and water molecule in the active site of CA XII is a likely reason for the decrease in entropy of the system.

There are three crystal structures of CA XIII with **4a**, **4b**, and **4c** (Fig. 10). The **4c** structure shows the formation of one hydrogen bond with a water molecule while **4b** – with three. This change may be the reason that causes the increase in the entropy as methyl and oxo groups are introduced in the pyrimidine ring.

It is difficult to make unambiguous correlations between the crystal structures and the thermodynamics of binding. However, several trends are clear. First, CA II structures with **3a**, **3b**, and **4b**, exhibit relatively small entropic contribution to binding (1.0 to 11.5 kJ/mol), while all remaining 6 compounds exhibit relatively large entropic contribution to binding (18.7 to 30.0 kJ/mol). This correlates with the fact that in three former structures, the second ring is in the same orientation and well represented by the electron density, while the latter 6 structures exhibit a different orientation of the second ring and are mostly poorly represented by the electron density. The latter 6 structures are less ordered and therefore there is larger portion of the entropic drive to the binding reaction.

## Conclusions

It is important to distinguish the observed thermodynamic parameters from intrinsic. Only the intrinsic parameters could be correlated with the structure. The intrinsic parameters of CA binding show that the incorporation of chlorine both in *meta* and *para* substituted compounds, increases the favorable entropic and decreases the favorable enthalpic contribution to the binding affinity (except in CA VII with *meta* substituted compounds). The *para* substituted compounds exhibit stronger affinities than *meta*. The addition of tert-butyl group, increases the affinity for CA I and CA XIII, but not for CA II, CA VII, and CA XII. All 16 tested compounds showed only partial additivity of thermodynamic parameters. All 16 compounds exhibited enthalpically-driven binding with a relatively small but variable entropic contribution. At least two independent methods that measure binding, such as FTSA and ITC, are required to accurately measure the observed affinities. Enthalpies are important for the determination of intrinsic thermodynamic parameters. The use of non-intrinsic observed parameters would lead to incomplete or inaccurate determination of the underlying forces and energetic reasons of binding needed for drug design.

Crystallographic data shows that substitutions in the pyrimidine ring considered in the present work do not influence the location of inhibitors in CA II. However, the differences in the position of substituents in the headgroup ring influence the position of inhibitor binding. The Cl atom in most cases is directed into the hydrophobic pocket. *Para*-substituted compounds **4**, despite variations in the position of the Cl-benzene, have two fixed conformations of the pyrimidine moiety regardless of the pyrimidine-substituents. The *meta*-substituted **1**, due to the steric clashes with the protein moiety, show variations in the positions of pyrimidine rings. Same trend is observed comparing the binding of **2** and **3** in CA II: *para*-substituted compounds have a similar way of binding, regardless of pyrimidine decorations, while *meta*-substituted compounds position pyrimidine rings in a different way due to steric clashes. Detailed correlation between the crystal structures and the enthalpies and entropies of binding largely confirm that enthalpic binders make tighter contacts and are more ordered in crystal structures, while compounds that are partially disordered in crystal structures have greater entropy contribution to the binding energetics. Therefore, enthalpy-driven compounds are better leads for drug design than entropy drive-compounds.

## Materials and Methods

### Chemistry

The synthesis, chemical structural characterization, and purity of the 16 compounds used in this study has been previously described by Čapkauskaitė et al. [12], [33]. Instant JChem was used for structure database management, search and prediction, Instant JChem 6.1.3, 2013, ChemAxon (http://www.chemaxon.com).

### Protein preparation

Expression and purification of human CA I, CA II, CA VII, CA XII, and CA XIII has been previously described: CA I by Baranauskienė et al. [38], CAII by Cimmperman et al. [39], CA VII and CA XIII by Sūdžius et al. [40], and CA XII by Jogaitė et al. [35].

### Fluorescent thermal shift assay (FTSA)

The binding parameters of 16 sulfonamide inhibitors were determined by the fluorescent thermal shift assay (FTSA). This method is used for rapid screening of protein and compound interactions[41]–[43]. FTSA measurements were performed in a Corbett Rotor-Gene 6000 (QIAGEN Rotor-Gene Q) instrument using the blue channel (excitation 365±20, detection 460±15 nm). Samples contained 5 µM protein, 0–200 µM compound, 50 µM solvatochromic dye ANS (8-anilino-1-naphthalene sulfonate) in 50 mM phosphate buffer (pH 7.0) containing 100 mM NaCl and the final DMSO concentration 2%. The applied heating rate was 1°C/min. Data analysis was performed as previously described by Baranauskiene et al. [38].

### Isothermal titration calorimetry (ITC)

Isothermal titration calorimetry (ITC) measurements of inhibitor binding to proteins were performed using VP-ITC instrument (Microcal, Inc., Northampton, USA) with 6–20 µM protein solution in the cell and 60–200 µM of the compound solution in the syringe. ITC instrument measures the heat evolution during the titration and can be used to determine the Δ*G*, Δ*H*, and Δ*S* of a reaction [44], [45]. A typical experiment consisted of 25 injections (10 µl each) added at 3 min intervals. Experiments were performed at 37°C in 50 mM phosphate buffer containing 100 mM NaCl at pH 7.0, with a final DMSO concentration of 2%, equal in the syringe and the cell. All experiments were repeated at least twice.

The enthalpy of inhibitor deprotonation was also measured using VP-ITC instrument. Inhibitor solution (0.25 mM) was deprotonated by adding 1.5 equivalent of NaOH and titrated with 2.5 mM nitric acid. A typical experiment consisted of 58 injections (5 µl each) added at 3 min intervals. Experiments were performed at 37°C with final DMSO concentration of 0.5%, equal in the syringe and the sample cell. All experiments were repeated at least three times.

### Stopped-flow kinetic CO_2_ hydration assay (SFA)

Enzyme inhibition experiments were performed using Applied Photophysics SX.18MV-R stopped-flow spectrometer at 25°C. Stopped-flow kinetic CO_2_ hydration assay is used for direct determination of carbonic anhydrase activity and inhibitory properties of compounds [46], [47]. Reaction velocities were measured by recording the absorbance of bromthymol blue indicator (50 µM, 615 nm). The sample consisted of 100–500 nM CA I, 20 nM CA II, 50 nM CA VII, 100 nM CA XII or 200–500 nM CA XIII, 0–10 µM **1b** inhibitor (in ≤0.2% DMSO) and MOPS buffer containing 100 mM NaCl, pH 7.0. Saturated CO_2_ solution was prepared by bubbling the CO_2_ gas in Milli-Q water at 25°C for 1 hour. A single exponential model was used to fit raw curves and the *IC_50_* was determined using sigmoidal dose-response model with the Hill slope of 1. The observed *IC_50_* of compound **1b** determined by SFA are compared with *K_d_* obtained by FTSA, ITC in Table 4.

**Table 4:** Comparison of *K_d_* (determined by ITC, FTSA) and *IC_50_* (determined by SFA) of the compound **1b** binding to five isoforms of human recombinant CAs.

	*K_d_*, nM (ITC)	*K_d_*, nM (TSA)	*IC_50_*, nM (SFA)
CAI	2300	3100	5550
CAII	430	330	220
CAVII	660	710	830
CAXII	370	1300	1100
CAXIII	270	330	450

All three methods provided the same values within the error margin.

### Measurement of pK_a_ of the sulfonamide group of the compounds

The p*K_a_* of the sulfonamide group of the compounds was measured with the spectrophotometer “Agilent 89090A” as previously described by Snyder et al. [34]. Ultraviolet spectra (λ = 200–380 nm) were collected at each pH between 6.0 and 12.0 at intervals of 0.5 pH units. The ratio of absorbances at two wavelengths (10 nm above and below isosbestic point) was plotted as a function of pH. The p*K_a_* value was determined as a midpoint of the curve. The p*K_a_* values were also calculated using Marvin Sketch software and confirmed by ACD/Labs online calculator I-Lab 2.0.

### Intrinsic thermodynamics

The observed binding constants and observed enthalpies are dependent on buffer components and pH. Observed parameters were experimentally measured in phosphate buffer at pH 7.0, 37°C. The intrinsic binding constant *K_b_* is equal to observed binding constant *K_b_obs_* divided by the available fractions of deprotonated inhibitor and protonated zinc hydroxide anion:
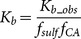
(1)


The fractions of deprotonated inhibitor and CA with the protonated water molecule bound to zinc in the active site can be calculated if both p*K_a_* values are known:
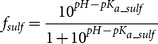
(2)




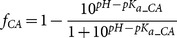
(3)


The observed enthalpy is the sum of all protonation events and the binding reaction. Linked reactions that have to be subtracted are the protonation/deprotonation of buffer, deprotonation of sulfonamide, and protonation of the hydroxide anion bound to active site zinc:
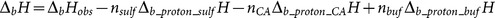
(4)where the intrinsic binding enthalpy is Δ*_b_H*, the observed binding enthalpy is Δ*_b_H_obs_*, enthalpy of inhibitor protonation – Δ*_b_proton_sulf_ H*, enthalpy of CA protonation – Δ*_b_proton_CA_ H,* the buffer protonation enthalpy – Δ*_b_proton_buf_ H, n_sulf_* = 1 − *f_sulf_, n_CA_* = 1 *− f_CA_* and *n_buf_ = n_sulf_+n_CA_*. *n_sulf_* is the number of protons released during the deprotonation of sulfonamide, *n_CA_* is the number protonated hydroxide ions bound to the active site Zn and *n_buf_* is the number of protons taken or released by the buffer.

### Crystallization

Human CA II and CA XIII in 20 mM NaHepes, pH 7.5 and 50 mM NaCl were concentrated to the concentration 20–50 mg/ml. Crystals were grown by sitting drop vapor diffusion method by mixing equal volumes of protein solution and crystallization buffer at 20°C. Buffers used for crystallization are presented in Table 5. Co-crystals of CA with inhibitors were prepared by soaking with 0.5 mM solution of inhibitor in crystallization buffer. Soaking solutions were prepared by mixing of 50 mM stock solution of inhibitor in DMSO with crystallization solution from reservoir. Soaking was performed several days before data collection.

**Table 5:** X-ray crystallographic data collection and refinement statistics.

Isoform-compound	CA II-4c	CA II-2c	CA II-1d	CA XIII-4c
PDB ID	4QSA	4QSB	4QSI	4QSJ
Crystallization buffer	2M sodium malonate (pH 7.5)	2.2M sodium malonate (pH 7.5)	0.1M sodium BICINE (pH 9.0) and 3.1M sodium malonate (pH 7.0)	0.1M sodium citrate (pH 5.5), 0.2M sodium acetate (pH 4.5) and 28% PEG4000
Spacegroup	P2_1_	P2_1_	P2_1_	P2_1_2_1_2_1_
Unit cell (Å)	a = 42.12, b = 40.99, c = 71.64, α = γ = 90°, β = 104.06°	a = 42.25, b = 40.98, c = 71.48, α = γ = 90°, β = 104.04°	a = 42.06, b = 40.92, c = 71.59, α = γ = 90°, β = 104.01°	a = 56.51, b = 57.68, c = 159.88, α = β = γ = 90.0°
Resolution (Å)	23.31–1.50	18.75–1.40	24.36–1.95	39.10–1.70
*N_ref_* (unique)	38075	46809	16250	57690
*R_merge_*, (outer shell)	0.065 (0.119)	0.099 (0.390)	0.048 (0.263)	0.060 (0.377)
*I/σ* (outer shell)	17.3 (8.5)	16.8 (2.5)	28.1 (6.3)	18.1 (3.8)
Multiplicity (outer shell)	7.0 (5.5)	5.0 (5.0)	6.8 (6.5)	5.2 (5.2)
Completeness (%) (outer shell)	99.2 (94.7)	99.7 (99.9)	93.6 (89.1)	99.2 (99.3)
*N_atoms_*	2475	2374	2292	4942
*R_work_*	0.166	0.188	0.157	0.166
*R_free_*	0.205	0.220	0.209	0.202
*B_average_*	15.138	16.507	19.510	17.382
*RMS_bonds_*	0.024	0.025	0.018	0.022
*RMS_angles_*	2.289	2.392	1.892	2.163

All datasets were collected at 100 K, test set size was 10%.

### Data collection and structure determination

All datasets except 4QSI were collected at the EMBL beam line X13 at the DORIS storage ring (DESY, Hamburg). 4QSI dataset was collected using X-ray diffractometer MicroMax 007 HF (Rigaku, Japan) equipped with RaxisIV++ detector at the Institute of Biotechnology (Vilnius, Lithuania). Datasets were processed using CCP4 program suite [48]: MOSFLM [49], TRUNCATE [50] and SCALA [51]. Structures were solved by molecular replacement using MOLREP [52]. The protein chain from the PDB entry 3HLJ was used as a starting model for CA II structures and PDB entry 2NN0 for CA XIII. Models were refined with REFMAC [53] and manually corrected in COOT [54]. Atomic coordinates of compounds were generated using molecular editor Avogadro [55]. Descriptions of compound geometry for model refinement were generated using LIBREFMAC [56]. The data collection and refinement statistics of PDB entries are presented in Table 4. ACCESSION NUMBERS: Coordinates and structure factors have been deposited in the Protein Data Bank with accession numbers 4QSA, 4QSB, 4QSI, and 4QSJ.

## Supporting Information

S1 Figure Inhibitor structure correlations with the observed and intrinsic affinity. Observed and intrinsic (in the brackets) Δ*_b_G* and *K_d_* values of compound binding to five investigated CA isoforms are given within the shapes. Different colors represent different CA isoforms. Numbers next to arrows show the Gibbs free energy (top number) and *K_d_* (bottom number) of binding differences between two neighboring compounds (Δ*_b_G* in kJ/mol at 37°C). Numbers to the top and right of the map are averages between same heads and tails of the compounds. The standard deviations indicate the presence or absence of the energetic additivity of compound functional groups. Note that observed and intrinsic affinities often differ by more than 100 fold.
(TIF)

S2 Figure Electron densities of compounds **1d**, **2c**, and **4c** in the active centers of CA II and CA XIII. Electron density maps |F_obs_-F_calc_| were calculated in the absence of compound. Alternative conformations of compounds are shown in different colors. Zn atoms are shown as blue spheres. A, Electron density map of **1d** bound to CA II and contoured at 2.8σ. B, Two conformations of **2c** modeled in the active center of CA II. Electron density map contoured at 2.3 σ. C, Two alternative positions of the second ring of **4c** modeled in CA II. Electron density map contoured at 2.5σ. D, Compound **4c** in the active center of CA XIII, protein chain B. Electron density map contoured at 2.7σ. E, Compound **4c** in the active center of CA XIII, protein chain A. Electron density map contoured at 2.6σ.
(TIF)
