# Supplementary figures and images for: Intrinsic Thermodynamics and Structure Correlation of Benzenesulfonamides with a Pyrimidine Moiety Binding to Carbonic Anhydrases I, II, VII, XII, and XIII
Source: PLoS One. 2014 Dec 10;9(12):e114106. doi: 10.1371/journal.pone.0114106 (PMC4262373; doi:10.1371/journal.pone.0114106)

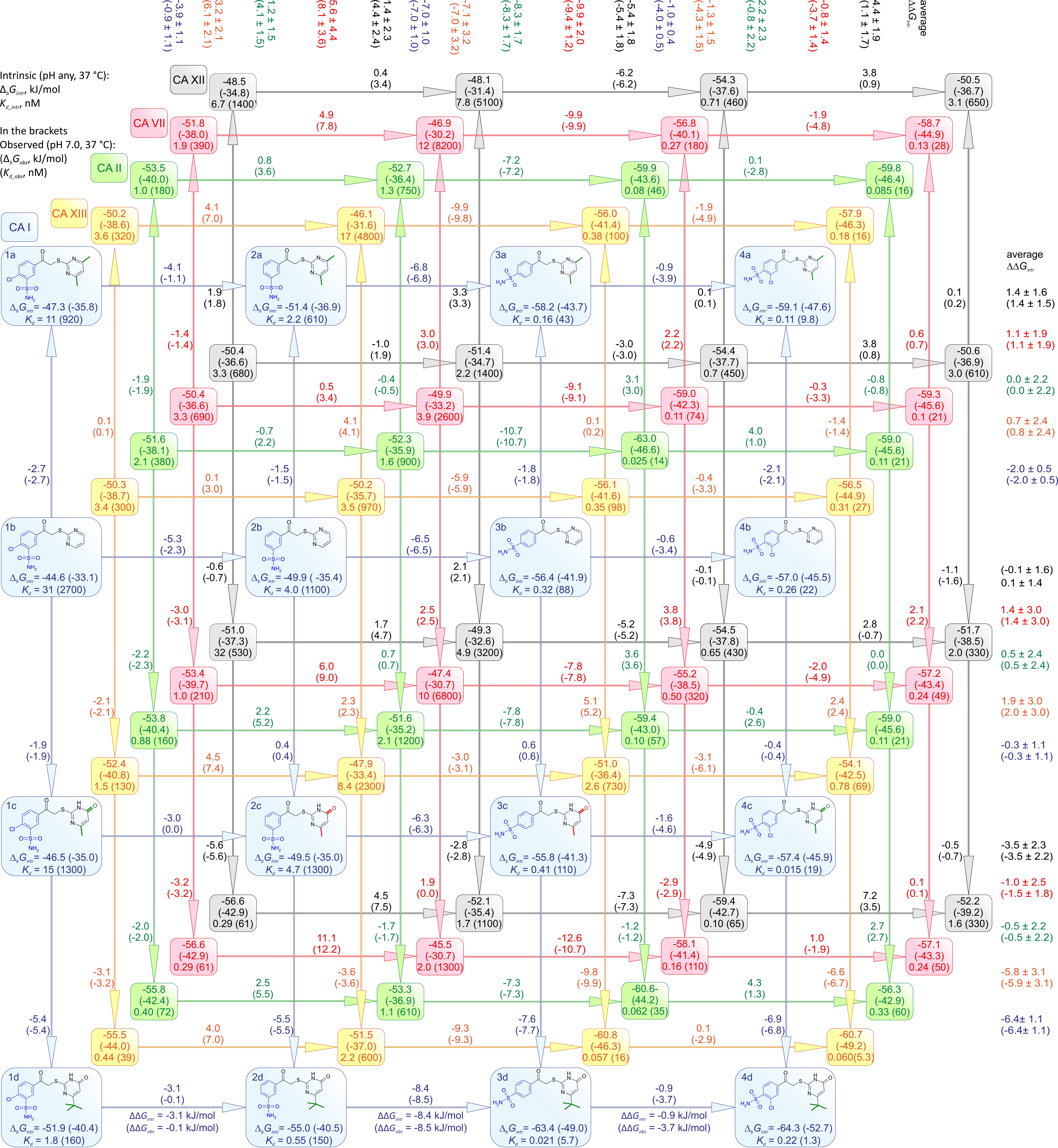

Supplement: S1 Figure — Inhibitor structure correlations with the observed and intrinsic affinity. Observed and intrinsic (in the brackets) ΔbG and Kd values of compound binding to five investigated CA isoforms are given within the shapes. Different colors represent different CA isoforms. Numbers next to arrows show the Gibbs free energy (top number) and Kd (bottom number) of binding differences between two neighboring compounds (ΔbG in kJ/mol at 37°C). Numbers to the top and right of the map are averages between same heads and tails of the compounds. The standard deviations indicate the presence or absence of the energetic additivity of compound functional groups. Note that observed and intrinsic affinities often differ by more than 100 fold. (TIF) [file pone.0114106.s001.tif]

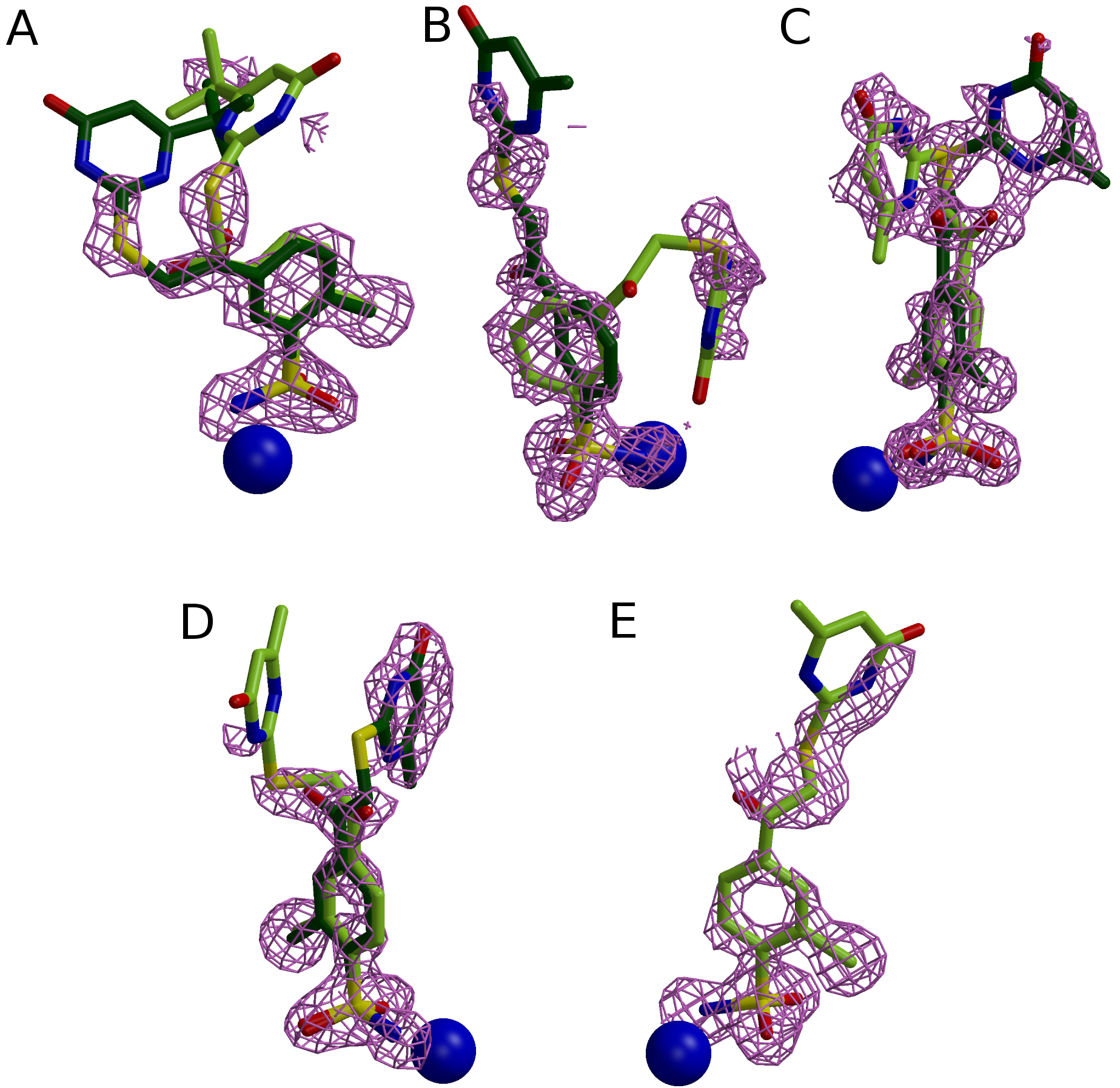

Supplement: S2 Figure — Electron densities of compounds 1d, 2c, and 4c in the active centers of CA II and CA XIII. Electron density maps |Fobs-Fcalc| were calculated in the absence of compound. Alternative conformations of compounds are shown in different colors. Zn atoms are shown as blue spheres. A, Electron density map of 1d bound to CA II and contoured at 2.8σ. B, Two conformations of 2c modeled in the active center of CA II. Electron density map contoured at 2.3 σ. C, Two alternative positions of the second ring of 4c modeled in CA II. Electron density map contoured at 2.5σ. D, Compound 4c in the active center of CA XIII, protein chain B. Electron density map contoured at 2.7σ. E, Compound 4c in the active center of CA XIII, protein chain A. Electron density map contoured at 2.6σ. (TIF) [file pone.0114106.s002.tif]
